# Ponte Osteotomies in the Surgical Treatment of Adolescent Idiopathic Scoliosis: A Systematic Review of the Literature and Meta-Analysis of Comparative Studies

**DOI:** 10.3390/children11010092

**Published:** 2024-01-12

**Authors:** Cesare Faldini, Giovanni Viroli, Matteo Traversari, Marco Manzetti, Marco Ialuna, Francesco Sartini, Alessandro Cargeli, Stefania Claudia Parisi, Alberto Ruffilli

**Affiliations:** 1 Department of Biomedical and Neuromotor Science—DIBINEM, University of Bologna, 40126 Bologna, Italy; giovanni.viroli@ior.it (G.V.); matteo.traversari@ior.it (M.T.); marco.manzetti@ior.it (M.M.); marco.ialuna@ior.it (M.I.); francesco.sartini@ior.it (F.S.); alessandro.cargeli@ior.it (A.C.); stefaniaclaudia.parisi@ior.it (S.C.P.); alberto.ruffilli@ior.it (A.R.); 2 1st Orthopaedic and Traumatologic Clinic, IRCCS Istituto Ortopedico Rizzoli, 40136 Bologna, Italy

**Keywords:** adolescent idiopathic scoliosis, AIS, Ponte osteotomies, posterior column osteotomies, deformity correction

## Abstract

The purpose of the present paper is to assess if Ponte osteotomies (POs) allow for a better correction in adolescent idiopathic scoliosis (AIS) surgery and to investigate their safety profile. A systematic search of electronic databases was conducted. Inclusion criteria: comparative studies that reported the outcomes of AIS patients who underwent surgical correction through posterior-only approach with and without POs. Clinical and radiographic outcomes were extracted and summarized. Meta-analyses were performed to estimate the differences between patients treated with and without POs. *p* < 0.05 was considered significant. In total, 9 studies were included. No significant difference in thoracic kyphosis (TK) change between patients treated with and without POs was found (+3.8°; *p* = 0.06). Considering only hypokyphotic patients, a significant difference in TK change resulted in POs patients (+6.6°; *p* < 0.01), while a non-significant TK change resulted in normokyphotic patients (+0.2°; *p* = 0.96). No significant difference in coronal correction (2.5°; *p* = 0.10) was recorded. Significant estimated blood loss (EBL) (142.5 mL; *p* = 0.04) and surgical time (21.5 min; *p* = 0.04) differences were found with POs. Regarding complications rate, the meta-analysis showed a non-significant log odds ratio of 1.1 (*p* = 0.08) with POs. In conclusion, POs allow for the restoration of TK in hypokyphotic AIS, without a significantly greater TK change in normokyphotic patients, nor a significantly better coronal correction. Considering the significantly greater EBL and the trend toward a higher complications rate, the correct indication for POs is crucial.

## 1. Introduction

Adolescent idiopathic scoliosis (AIS) surgery, during the past 20 years, has experienced major advancements. More specifically, the wide spread of modern pedicle fixation systems, along with the development of powerful corrective techniques such as direct vertebral rotation (DVR), has enabled powerful posterior-only corrective surgeries, especially in the coronal and axial planes. Conversely, considering the tridimensional nature of AIS, the results of surgical correction on the sagittal plane component of the deformity, typically characterized by a reduction in thoracic kyphosis (TK) due to anterior spinal overgrowth [1], have been inconsistent [2]. In particular, several studies have demonstrated not only a failure in the restoration of TK, but also a proper iatrogenic hypokyphotic effect, which has been ascribed to DVR at times [3,4,5] and to all-pedicle-screws-based constructs [6,7]. This aspect of AIS surgery has received growing attention since thoracic hypokyphosis is related to long term consequences in the adjacent spinal regions. In particular, Bernstein et al. [8] reported an increased risk of lumbar degenerative disc disease in patients in which TK restoration was ineffective, while Hwang et al. [9] correlated the lack of TK restoration to an increased risk of cervical spine decompensation in kyphosis after AIS surgery.

Many authors [10,11] have therefore adopted ancillary procedures such as Ponte osteotomies (POs) in order to restore TK, or at least to optimize the corrective maneuver, trying to avoid the risk of iatrogenic hypokyphosis as much as possible. Ponte osteotomies were first developed in 1987 by Alberto Ponte for the surgical correction of rigid hyperkyphosis [12]. In particular, the original technique requires a wide multilevel posterior release with the removal of all posterior column ligaments, a superior and inferior laminectomy, and a bilateral extended facetectomy. This results in substantial posterior column shortening when the osteotomy is closed. However, scoliosis correction requires an opposite effect: an elongation of the posterior column in order to restore TK in hypokyphotic scoliosis or to avoid iatrogenic hypokyphosis in normokyphotic curves. This conceptual contradiction has added to the scepticism regarding the efficacy of POs in scoliosis surgery, especially considering that POs are not risk-free.

Through a systematic literature research and a meta-analysis of comparative studies, the first aim of the present paper is to assess whether the adoption of POs allows for the restoration of TK during AIS correction surgery. The second objective is to assess the influence of POs on the coronal correction rate. The final endpoint is to determine if the use of POs results in significantly increased blood loss, operative time, and complication rate, such that their adoption for a better correction may not be justified by their safety profile.

## 2. Materials and Methods

A systematic review of the literature regarding the effect of POs on thoracic kyphosis as accessory procedures of surgical treatment of AIS was conducted in accordance with the PRISMA guidelines (preferred reporting items of systematic reviews) [13].

### 2.1. Eligibility Criteria

Only peer-reviewed publications were considered for inclusion. Studies were included if they compared the outcomes of patients affected by AIS who underwent surgical correction through a posterior-only approach with and without Ponte osteotomies. Articles in English which met the PICO (Population, Intervention, Comparison, and Outcomes) criteria on systematic reviews were considered for inclusion.

Only randomized controlled trials (RCTs) and prospective and retrospective comparative cohort studies (PCS and RCS) were considered for inclusion. In vitro studies and animal model studies were excluded, as well as case reports and case series.

### 2.2. Search Strategy

Studies eligible for this systematic review were identified through an electronic systematic search of PubMed and Cochrane Central Registry of Controlled Trials papers published from 2000 to May 2023.

The following search strings were used:

- (adolescent AND idiopathic AND scoliosis) OR (AIS) AND (ponte OR (ponte AND osteotomy) OR (ponte AND osteotomies) OR (multiple AND asymmetric AND ponte AND osteotomies) OR MAPO);
- ((scoliosis AND adolescent) OR AIS)) and (ponte OR (ponte AND osteotomy) OR (ponte AND osteotomies) OR MAPO OR (posterior AND column AND osteotomies) OR (posterior AND column AND osteotomy) OR (PCO)).

### 2.3. Study Selection

**Figure 1:**
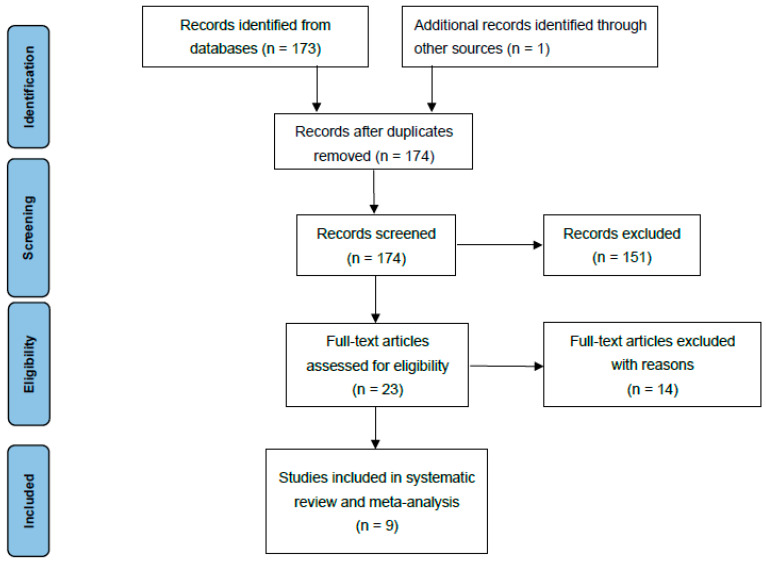
Prisma flowchart.

Articles considered relevant by electronic search were retrieved in full-text, and a hand-search of their bibliography was performed in order to find further related articles. Reviews and meta-analyses were also analysed to identify potentially missed eligible papers. Duplicates were removed. The study selection process was carried out in accordance with the PRISMA flowchart (Figure 1). The systematic review was not prospectively registered.

**Figure 2:**
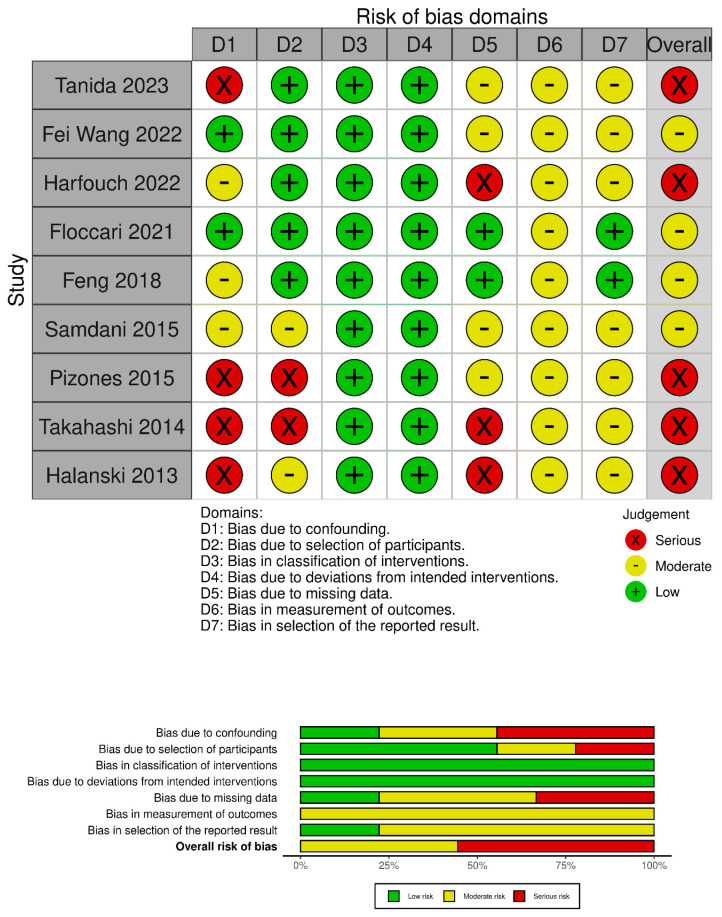
Risk of bias assessment according to Robins-I tool [15,16,17,18,19,20,21,22,23].

The quality of the included studies was evaluated using the Robins-I tool [14] (Figure 2).

### 2.4. Data Collection Process

All included studies were analysed, and data related to baseline characteristics (Table 1) and outcomes of interest (Table 2) were extracted and summarized.

Meta-analyses were performed when there were at least three studies to be compared. Heterogeneity between studies was assessed using the inconsistency statistic (I^2^ > 75% was considered to be high heterogeneity). Publication bias was assessed with Egger’s test and represented with forest plots. Standardized mean differences were used as measures of effect size. The random effect model was applied. A *p*-value < 0.05 was considered to be significant. All statistical analyses were conducted with Jamovi version 2.2 (The Jamovi project, Sydney, Australia) software.

## 3. Results

### 3.1. Baseline Studies Characteristics and Quality Assessment

A total of 174 studies were found through electronic search; after screening, 9 studies (1 prospective comparative matched cohort study (PCS), 1 historically controlled cohort study (HCCS), and 7 retrospective comparative cohort studies (RCS) were included. Meta-analysis was conducted on comparative studies. The risk of bias in the papers is reported in Figure 2.

A total of 667 patients were included. The mean age at surgery ranged from 13.2 ± 3.0 [15] to 16.7 ± 3.4 years [16]. Lenke type was reported for 486 patients: 353 Lenke 1 (72.6%), 87 Lenke 2 (17.9%), 20 Lenke 3 (4.1%), 10 Lenke 4 (2.1%), and 16 Lenke 6 (3.3%). As for constructs, 8 authors used all pedicle screws constructs [15,16,17,18,19,20,21,22], while one preferred hybrid constructs [23]. As for Ponte osteotomies, most authors performed a variable number of periapical osteotomies ranging from 2 to 9 [18].

### 3.2. Thoracic Kyphosis Change

**Figure 3:**
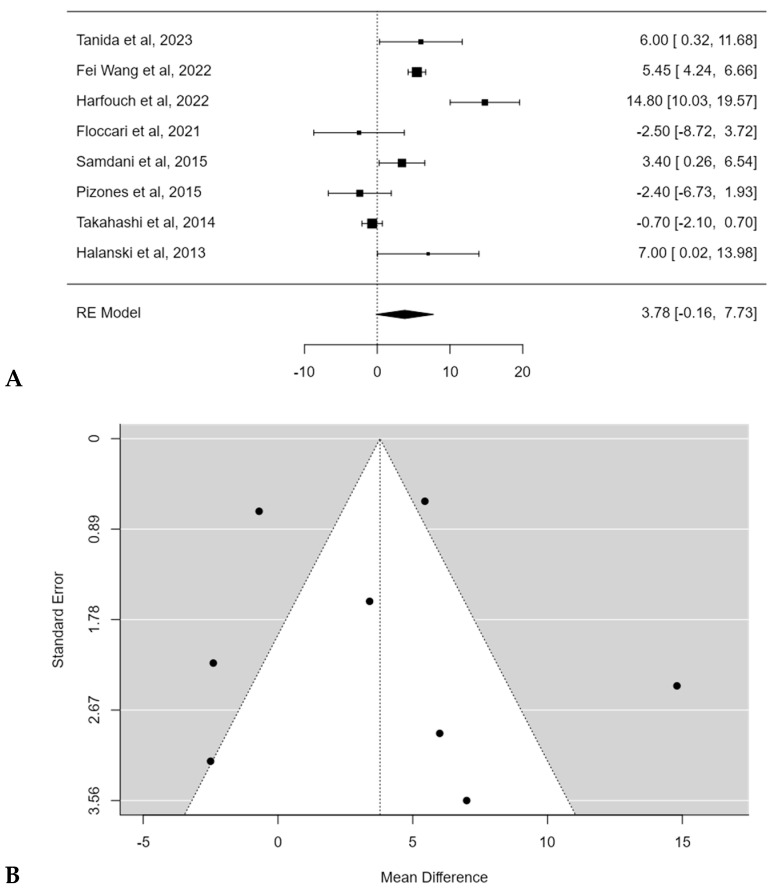
Forest plot (**A**) and funnel plot (**B**) of thoracic kyphosis change difference in meta-analysis between groups treated with and without POs [15,16,17,18,20,21,22,23].

The mean pre-operative thoracic kyphosis (T5–T12) varied from 5.3 ± 3.2° [17] to 36.2 ± 14.9° [16]. The mean thoracic kyphosis change after surgery ranged between −5.5° [18] to 18.9° [17] for POs groups and from −18.6° [16] to 13.5° [17] after posterior spinal fusion (PSF) without POs. Harfouch et al. [16] reported a larger difference in TK between patients treated with and without POs, reporting 3.8° of TK loss after PSF with POs and 18.6° of kyphosis loss after PSF without POs (*p* < 0.001). No significant difference in TK change between PSF with and without POs was found in the meta-analysis, with an estimated average mean difference of +3.8° (95% CI: −0.1644 to 7.7319°; *p* = 0.0603) (Figure 3A). No publication bias (t = 0.393, *p* = 0.694 (Figure 3B) was found, but substantial heterogeneity among studies was calculated (I^2^ = 93.5303%, *p* < 0.0001).

**Figure 4:**
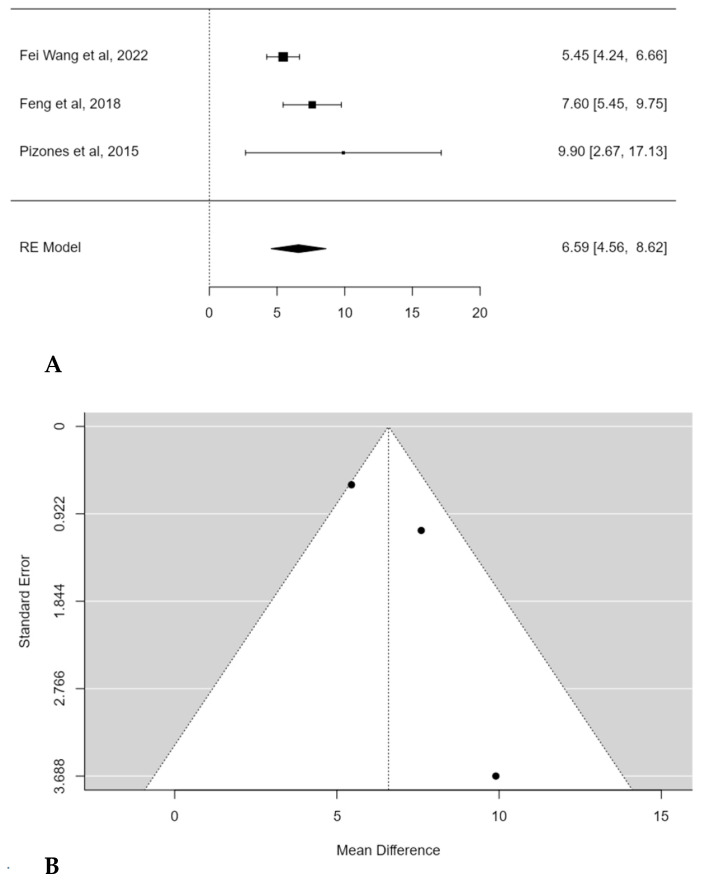
Forest plot (**A**) and funnel plot (**B**) of thoracic kyphosis change difference in meta-analysis between groups treated with and without POs, considering only hypokyphotic patients [17,19,23].

A subgroup meta-analysis was performed in order to assess the mean TK change considering only hypokyphotic patients (Lenke sagittal modifier = TK < 10°). In this, three studies were included, and POs provided a significant estimated average TK increase in this subset of patients (+6.6°; 95% CI: 4.5586 to 8.6236; *p* < 0.0001) (Figure 4A). No publication bias was found (t = 1.560; *p* < 0.119), nor was significant heterogeneity detected (I^2^ = 52.3883%, *p* = 0.1331) (Figure 4B).

**Figure 5:**
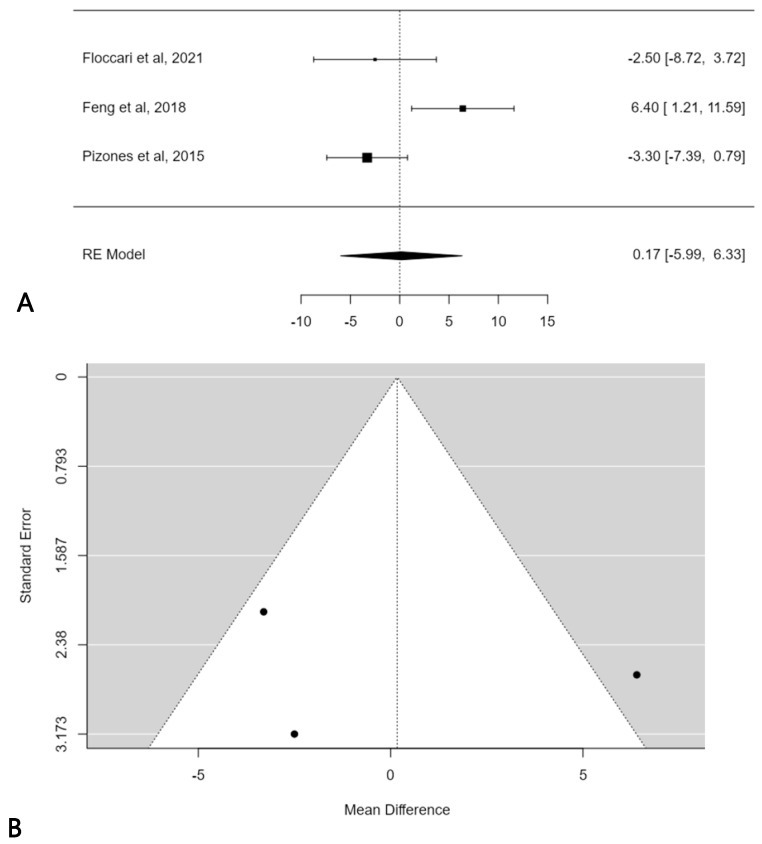
Forest plot (**A**) and funnel plot (**B**) of thoracic kyphosis change difference in meta-analysis between groups treated with and without POs, considering only normokyphotic patients [18,19,23].

A further subgroup meta-analysis was performed in order to assess the mean TK change considering only normokyphotic patients (Lenke sagittal modifier N = 10° < TK < 40°). In this, three studies were included, and the estimated average mean TK change difference was 0.2° (95% CI: −5.9864 to 6.3258) without statistical significance (*p* = 0.9569) (Figure 5A). No publication bias was found (t = 0.121; *p* < 0.903), but significant heterogeneity was detected (I^2^ = 76.9945%, *p* = 0.0114) (Figure 5B).

### 3.3. Coronal Deformity Correction Rate

The mean Cobb angle of the major curve varied from 48.1 ± 3.9° [17] to 74.5 ± 15.2° [18], with a flexibility index ranging between 31.7% [21] and 54.5% [20]. Coronal correction rate of the major curve ranged between 62.0% [21] and 84.0% [15] in Pos groups, and from 58.7% [18] to 83.0% [15] in non-Pos groups.

**Figure 6:**
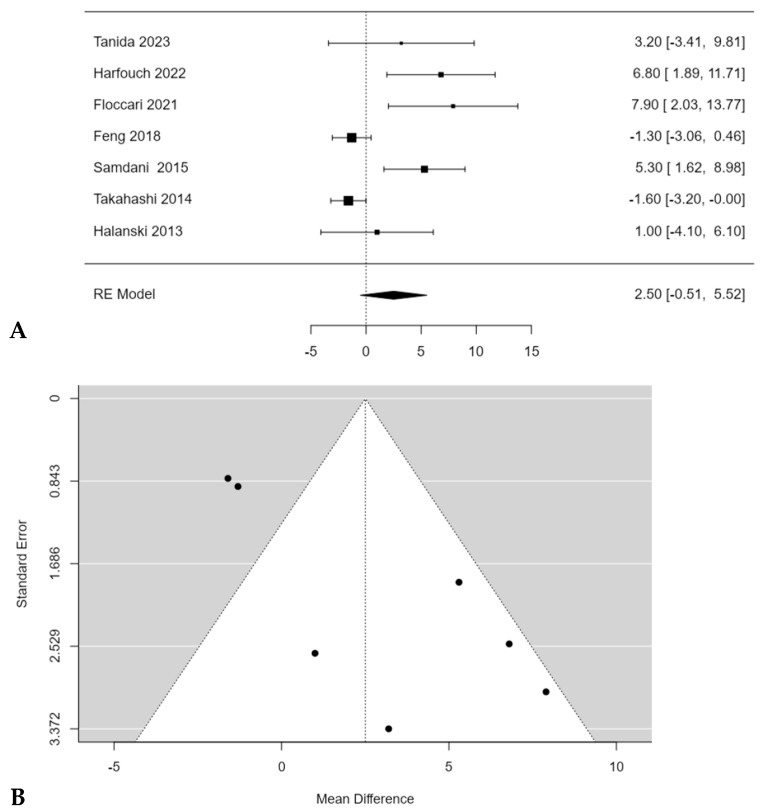
Forest plot (**A**) and funnel plot (**B**) of coronal correction rate difference in meta-analysis between groups treated with and without POs [15,16,18,19,20,21,22].

Floccari et al. [18] reported the largest difference in the coronal correction rate of the major curve Cobb angle between Pos and non-Pos group, reporting 66.6% coronal correction with Pos and 58.7% without Pos (*p* < 0.05). No significant difference in coronal correction with and without Pos was found during the meta-analysis (2.5%; 95% CI: −0.5118 to 5.5179; *p* = 0.1037) (Figure 6A), despite most of the studies reported higher correction rates in patients who underwent PSF with POS [15,16,18,20,22]. Feng et al. [19] and Takahashi et al. [21] reported a higher correction rate in patients who underwent PSF without Pos. A significant publication bias (t = 4.65, *p* < 0.001) was found, and a moderate inconsistency among studies was found too (I^2^ = 82.7373%; *p* < 0.0001) (Figure 6B).

### 3.4. Surgical Time and Blood Loss

The mean surgical time ranged from 236.0 [21] to 368.2 [22] minutes with Pos, and from 187.0 [21] to 339.8 [22] minutes without Pos. Takahashi et al. [21], Feng et al. [19], and Fei Wang et al. [17] reported significantly higher surgical times in patients treated with POs (*p* = 0.003, *p* < 0.001 and *p* < 0.001, respectively).

**Figure 7:**
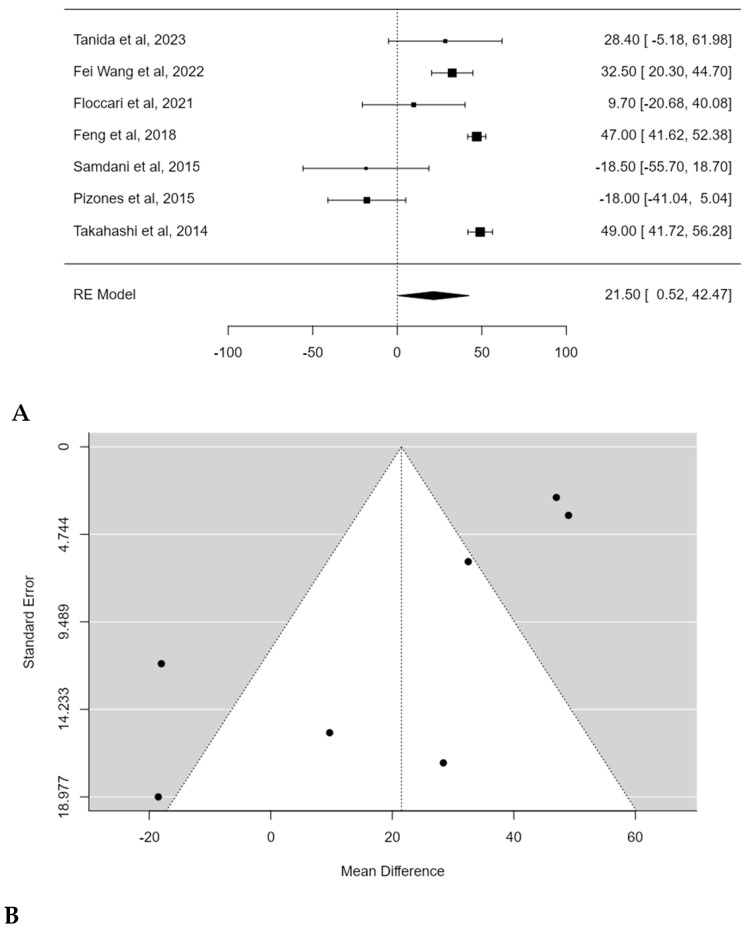
Forest plot (**A**) and funnel plot (**B**) of surgical time difference in meta-analysis between groups treated with and without POs [17,18,19,20,21,22,23].

A significant difference in surgical times between PSF with and without POs was found in the meta-analysis, with an estimated average mean difference of 21.5 min (95% CI: 0.5182 to 42.4744; *p* = 0.0446) (Figure 7A). Publication bias was found (t = −3.122, *p* = 0.002) and a substantial heterogeneity among studies was also revealed (I^2^ = 94.4656%, *p* < 0.0001) (Figure 7B).

The mean estimated blood loss (EBL) ranged from 619.7 [22] to 1141.0 mL [21] for patients treated with POs, and from 723.0 [22] to 979.8 mL [17] for patients treated without POs. Most authors reported significantly higher EBL in patients treated with POs [17,19,20,21], while Floccari et al. [18] and Tanida et al. [22] reported no significant differences in EBL between the two groups (*p* = 0.825 and *p* = 0.28, respectively).

**Figure 8:**
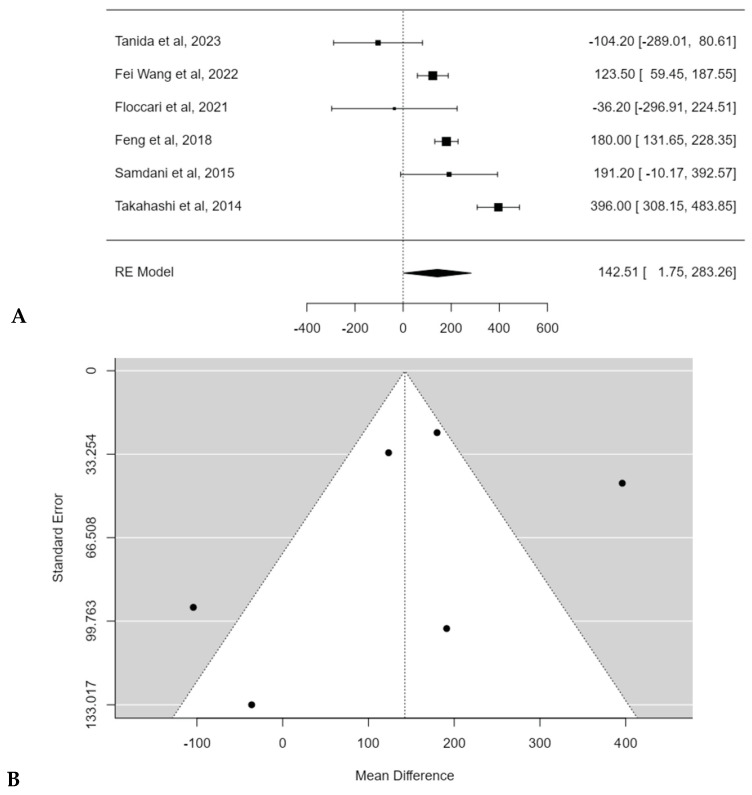
Forest plot (**A**) and funnel plot (**B**) of EBL difference in meta-analysis between groups treated with and without POs [17,18,19,20,21,22].

Meta-analysis confirmed the statistical significance of estimated average mean EBL difference between PSF with and without Pos: 142.5 mL (95% CI: 1.7474 to 283.2643; *p* = 0.0472) (Figure 8A). No publication bias was found (t = −1.291, *p* = 0.197), but a substantial heterogeneity among studies was discovered (I^2^ = 91.7023%; *p* < 0.0001) (Figure 8B).

### 3.5. Complications

The complications rate ranged from 3.1 [19] to 34.2% [18] for patients treated with POs, and from 0 [16] to 6.1% [18] for patients treated without POs. Most authors reported higher complications rate in patients treated with POs [16,17,18], while Feng al. [19] and Halanski et al. [15] reported a similar complications rate between the two groups. Complications were reported and stratified according to the modified Clavien–Dindo–Sink classification [24] in Table 3.

**Figure 9:**
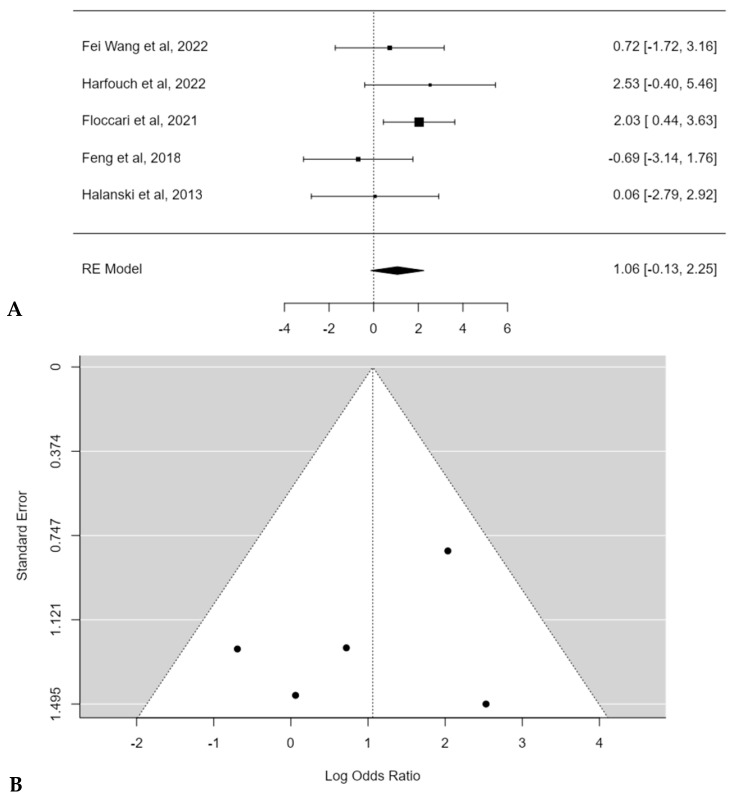
Forest plot (**A**) and funnel plot (**B**) of complications rate difference in meta-analysis between groups treated with and without POs [15,16,17,18,19].

The meta-analysis reported an estimated average log odds ratio of 1.1 (95% CI: −0.1272 to 2.2511) with POs, which was not statistically significant (*p* = 0.0801) (Figure 9A). No publication bias (t = −0.200, *p* = 0.817) or substantial heterogeneity among studies was found (I^2^ = 22.1072%; *p* = 0.3) (Figure 9B).

## 4. Discussion

When considering all the eligible studies, the present work showed that the use of POs does not lead to a significant difference in TK change after AIS correction, with an estimated average mean difference of +3.8° (95% CI: −0.1644 to 7.7319°; *p* = 0.0603).

However, when performing a subgroup meta-analysis including only hypokyphotic patients, the use of POs led to a significantly greater TK increase (+6.6°; *p* < 0.0001). These results must be seen in light of some important considerations. In fact, POs should just be considered as release procedures that improve spinal flexibility, allowing for the surgeon to shape the desired final sagittal profile of the spine more efficiently. In that view, in hypokyphotic patients, POs may allow for a more efficient posterior translation of the spine to the over-contoured concave rod, reducing, on one side, the risk of rod deformation and, on the other side, reducing the risk of screws’ pull-out. In addition to its statistical significance, it is crucial to determine if the difference in TK change achieved with the use of POs may also be clinically significant in hypokyphotic patients. In this regard, although slight when considered in absolute terms (+6.6°), the difference is actually remarkable when considered relative to the starting TK value (<10°), although we still do not know if this TK change may produce a true clinical difference. However, it must be noted that TK measurement can be challenging, particularly in AIS patients due to axial rotation and frontal inclination of vertebral bodies, with a reported measurement error of 6.68° (95% confidence interval 5.74–7.61°) [25]. In this view, apart from its unknown clinical significance, the estimated average TK increase of 6.6° may not overcome the measurement error.

An additional subgroup analysis was conducted considering only normokyphotic patients (TK between 10° and 40°), with an estimated average TK change difference of 0.2° (95% CI: −5.9864 to 6.3258; *p* = 0.9569). Although few studies were eligible, in this subset of patients, the use of POs did not seem to be supported by a better control of TK. In fact, interestingly, two studies [18,23] noticed a better TK control without POs. The possible explanation of this result is complex. In normokyphotic patients, the thoracic spine does not need a powerful posterior translation since TK is supposed to be physiologic. In these patients, TK should be preserved by a thoroughly performed corrective manoeuvre, alongside an accurate concave rod contouring. If DVR is inappropriately performed, exerting a pushing effect on the convex side, and if rods contouring is not adequate, a TK flattening may be seen. In this situation, the adoption of POs, due to their release effect, may even worsen the flattening effect generated by an incorrect deroto-translation.

Regarding the coronal correction rate, the use of POs did not lead a to a significant difference (2.5%; 95% CI: −0.5118 to 5.5179; *p* = 0.1037). However, it must be noted that the average coronal Cobb angle of the studies included in the analysis ranged between 50.8–74.5° and that the flexibility index ranged between 31.7 and 54.5%. Therefore, this result seems to apply only to non-severe, non-stiff, coronal deformities. Conversely, some authors [11,26] adopted multiple asymmetric POs for the management of severe (>90°) and stiff (flexibility index < 25%) curves. Unfortunately, these were not comparative studies, so they were not suitable for the present analysis. This may reflect an underlying selection bias, since surgeons who are comfortable with this kind of osteotomy would typically use them as an alternative to tricolumn-osteotomies when addressing severe and stiff curves in order to have a more powerful coronal translation and perform selective apical convex compression.

Regarding surgical time, the analysis showed a significantly longer operation time of 21.5 min (95% CI: 0.5182 to 42.4744; *p* = 0.0446) for POs groups. It is uncertain if such a mild difference in surgical time may be clinically significant, However, the fact that that many confounding factors could play a role must be considered (average fused levels, average implant density, average number of POs). Conversely, EBL was significantly greater in the POs groups (142.5 mL; 95% CI: 1.7474 to 283.2643; *p* = 0.0472). This result is not unexpected and it is in accordance with many previous studies [27,28]. Whether this difference in EBL may play a significant role in the clinical outcomes and in the risk of transfusion is still uncertain, especially considering that many factors can help reduce blood loss (tranexamic acid [29], ∊-aminocaproic acid [30], topical haemostatic agents [31]). Finally, we looked at the complications rate of POs. The meta-analysis reported an estimated average log odds ratio of 1.1 (95% CI: −0.1272 to 2.2511), which was not statistically significant (*p* = 0.0801). In addition to generic complications like mechanical failures or surgical site infections, which were represented in both groups, interestingly, two authors [16,18] reported 5 cases each of intraoperative neuromonitoring (IONM) changes in their POs groups. Many studies [32,33,34] related this specific complication to POs. This is difficult to interpret since many factors have an influence on IONM. On the metabolic side, the increased blood loss resulting from POs may lead to a spinal cord hypoperfusion and subsequent IONM change. On the mechanical side, the elongation of the posterior column in hypokyphotic patients, resulting from a distraction manoeuvre at the osteotomies sites, in addition to the elongation of the spine due to the correction of the coronal deformity, may overstretch the spinal cord.

The present study does not come without limitations. Firstly, many of the included studies did not have TK restoration as the main goal, which certainly may have led to a selection bias. Moreover, only a few studies performed a separate analysis of TK change in hypo-, normo- and hyperkyphotic patients. There is also a possible issue in the measurement method of TK since its measurement on plain X-rays can be extremely difficult in AIS patients, even when measured between T5 and T12. This in part due to an overshadowing effect by native thoracic anatomy and in part due the axial plane rotation of the vertebral bodies, which does not allow for a true lateral view with a 2d imaging [35,36]. This could be overcome by the adoption of 3D imaging TK measurement but, unfortunately, none of the included studies adopted such a measurement method. Moreover, the lack of 3D imaging analysis may underestimate the tridimensional corrective effect achieved with POs. This is exacerbated by the fact that it was not possible to conduct an axial plane meta-analysis, since only three studies reported axial plane corrections, with heterogeneous methods (two papers adopted scoliometer [18,20], one paper adopted CT scan [22]). Furthermore, the included studies were heterogeneous for what concerns baseline characteristics of the included patients. In particular, many studies comprised patients with different Lenke patterns: Harfouch et al. [16], Floccari et al. [18], Feng et al. [19], Pizones et al. [23], and Takahashi et al. [21] included double (III and VI) or triple curves (IV); meanwhile, all patients included by Tanida et al. [22], Fei Wang et al. [17], Samdani et al. [20], and Halanski et al. [15] had thoracic patterns (I or II). This may be a source of bias, since thoracic patterns tend to have less TK, while double and triple curves are more frequently normokyphotic. Moreover, only one study matched POs and non-POs cohorts; this may inevitably raise the variability between the patients in the POs and non-POs groups in each of the included studies. More crucially, many additional surgical factors with a possible influence on coronal and/or sagittal correction should have been more specially taken into account by the included studies. Specifically, pedicle screw density was specified just in two papers [18,21]; number of POs was specified by most [16,17,18,20,21,22] but not all authors [15,19,23]; rod material and diameter were not reported by two authors [19,20]. It should be further considered that the studies that reported rod material and diameter were highly heterogeneous in terms of their choices: some adopted 5.5 mm cobalt-chrome [16,17], some 6.35 stainless steel [23], and some hybrid choices (6 mm cobalt-chrome in concavity + 6 mm titanium alloy in convexity [22]). Moreover, some authors even adopted different rod choices among their cohorts of patients [15,18,21] and this may consequently account for some of the differences in the correction outcomes between POs and non-POs groups among these studies. Although screw density, number of Pos, and rod choice may be important factors in AIS surgical correction outcomes, they must be viewed as tools in the surgeon’s hands. In fact, particularly regarding sagittal plane restoration, in a multicenter study by Monazzam et al. [37], the only significant predictor of TK restoration was the surgeon. This emphasizes the importance of the intraoperative corrective technique, especially in terms of rod contouring and regarding how and which corrective forces are applied by the surgeon. Despite that, many of the included papers did not provide an accurate technique description [15,18,21], and one paper was a multicenter study [20] with an inevitable heterogeneity in surgical technique.

Finally, all the included studies had a retrospective, non-randomized design, which may possibly represent an additional source of bias. Despite that, this is the first meta-analysis on the highly debated topic regarding TK control with the use of POs during AIS surgery, and the fact that only comparative studies were included helped to keep the internal variability of each study as low as possible. Further comparative studies, with better stratified patients according to preoperative TK and more precise measurement methods, will further shed a light on this topic.

## 5. Conclusions

Ponte osteotomies allow for significant restoration of TK in hypokyphotic AIS curves, without a significantly greater TK change in normokyphotic patients. On the coronal plane, a significantly greater correction rate was not reported, despite the included studies not focusing on severe and/or stiff curves. Considering the significantly greater EBL and the trend toward a higher complications rate, it appears clear that the correct indication of POs is crucial. Particularly in hypokyphotic patients, the benefits of TK restoration may overcome the risks. Conversely, the routinary use of POs in non-severe, non-stiff, and normokyphotic curves should be discouraged.

## Figures and Tables

**Table 1:** Summary of baseline characteristics of the included studies. (AIS: adolescent idiopathic scoliosis; POs: Ponte osteotomies; PSF: posterior spinal fusion).

Author	Study Design	Level of Evidence	Patients N° (M/F)	Inclusion Criteria	Mean Patient Age (Years)	Lenke Types	Mean Risser Sign	Internal Fixation System	Posterior Release	Number of POs
Tanida 2023 (P) [22]	Retrospective comparative cohort study	III	19 (3/16)	AIS patients with Lenke 1 or 2 patterns, who underwent PSF	15.4 ± 2.8	I: 6 II: 13	/	Pedicle screws and rods	T6/7, 7/8, and 8/9 Ponte osteotomies	3
Tanida 2023 (C) [22]			18 (4/14)		15.6 ± 2.2	I: 7 II: 11	/			0
Fei Wang 2022 (P) [17]	Retrospective comparative cohort study	III	40 (4/36)	AIS patients < 18 years old, main thoracic curve > 40°, thoracic kyphosis (T5–T12) < 10°	14.50 ± 1.77	I: 40 (IA: 26, IB: 10, IC: 4)	3.80 ± 0.8	Pedicle screws and rods	Peri-apical Ponte osteotomies	3
Fei Wang 2022 (C) [17]			40 (2/38)		15.13 ± 1.57	I: 40 (IA: 18, IB: 16, IC: 6)	4.00 ± 0.8		/	0
Harfouch 2022 (P) [16]	Retrospective comparative cohort study	III	40 (8/32)	Consecutive patients affected by AIS and treated with PSF	16.7 ± 3.4	I: 14 II: 12 III: 7 IV: 5 V: 0 VI: 2	/	Pedicle screws and rods	Peri-apical Ponte osteotomies	4–6
Harfouch 2022 (C) [16]			40 (6/34)		16.1 ± 2.6	I: 15 II: 12 III: 5 IV: 4 V: 0 VI: 4	/		/	0
Floccari 2021 (P) [18]	Prospective comparative matched cohort study	III	34 (8/26)	AIS patients treated with PSF and receiving at least 2 POs, matched with analogous group who underwent PSF without the use of POs	14.6 ± 2.3	I: 11 (IA: 6, IB: 5) II: 16 (IIA: 13, IIB: 2, IIC: 1) III: 4 (IIIC: 4) VI: 3	/	Pedicle screws and rods	Peri-apical Ponte osteotomies	3.5 (2–9)
Floccari 2021 (C) [18]			34 (8/26)		14.8 ± 2.0	I: 11 (IA: 6, IB: 5) II: 16 (IIA: 13, IIB: 2, IIC: 1) III: 4 (IIIC: 4) VI: 3	/		/	0
Feng 2018 (P) [19]	Retrospective comparative cohort study	III	32 (10/22)	AIS patients affected by Lenke 1–4 curves treated with PSF	15.1 ± 1.9	Lenke types 1–4, details not mentioned	3.30 ± 0.90	Pedicle screws and rods	Multilevel Ponte osteotomies (at each segment of the thoracic curve)	/
Feng 2018 (C) [19]			33 (10/23)		15.7 ± 1.9		3.10 ± 1.0		Soft tissues release	0
Samdani,2015 (P) [20]	Retrospective comparative cohort study	III	125 (27/98)	Prospective consecutive patients affected by Lenke 1A and 1B curves treated with PSF with at least 2 years of follow up	14.8 ± 2.3	I: 127 (IA: 91, IB:34)	/	Pedicle screws and rods	Ponte osteotomies	4.3 ± 1.5
Samdani, 2015 (C) [20]			66 (10/56)		14.6 ± 2.1	I: 56 (IA: 50, IB: 16)	/		Partial facetectomies with removal of inferior articular facets	0
Pizones 2015 (P) [23]	Historically controlled cohort study	III	43 (9/34)	Prospective series of patients affected by thoracic AIS who underwent PSF with POs compared with historical series of patients who underwent PSF alone	14.9 ± 2.1	I-IV (not mentioned details)	/	Sublaminar wires and hybrid instrumentation	Ponte osteotomies	/
Pizones 2015 (C) [23]			30 (5/25)		15.2 ± 2.3	I-IV (not mentioned details)	/		Not mentioned	0
Takahashi 2014 (P) [21]	Retrospective comparative cohort study	III	17 (0/17)	Patients affected by AIS who underwent skip pedicle screw fixation with POs compared with patients who underwent skip pedicle screw fixation	15.6 ± 2.0	I: 11 II: 3 IV: 1 VI: 2	/	Skip pedicle screw fixation and rods	Ponte osteotomies	3.8 ± 1.3
Takahashi 2014 (C) [21]			21 (0/21)		14.4 ± 2.5	I: 15 II: 4 VI: 2	/		Partial facetectomies with removal of inferior articular facets	0
Halanski 2013 (P) [15]	Retrospective comparative cohort study	III	17 (5/12)	Consecutive patients affected by AIS with Lenke I or II curves treated with PSF	13.2 ± 3.0	/	/	Pedicle screws and rods	Ponte osteotomies	/
Halanski 2013 (C) [15]			18 (2/16)		13.7 ± 2.0	/	/		Partial facetectomies with removal of inferior articular facets	0

**Table 2:** Summary of reported outcomes of the included studies. (POs: Ponte osteotomies; TK: thoracic kyphosis; IONM: intraoperative neuromonitoring; PSF: posterior spinal fusion).

Author	Mean Pre-Operative Major Cobb Angle (°)	Mean Flexibility Index of Major Curve (%)	Mean Post-Operative Major Cobb Angle (°)	Mean Correction Rate (%)	Mean Pre-Operative TK (°)	Mean Post-Operative TK (°)	Mean TK Change (°)	Mean Surgical Time (min)	Mean Intraoperative Blood Loss (mL)	Mean Length of Stay (Days)	Complication Rate (%)
Tanida 2023 (P) [22]	59.7 ± 10.3	45.5 ± 10.1	21.0 ± 6.2	64.4 ± 10.2	17.3 ± 12.7	30.7 ± 6.4	13.8 ± 9.6	368.2 ± 54.5	619.7 ± 288.0	/	/
Tanida 2023 (C) [22]	53.1 ± 5.8	38.7 ± 15.9	20.6 ± 5.3	61.0 ± 10.3	11.6 ± 10.5	22.3 ± 4.7	7.8 ± 8.0	339.8 ± 49.7	723.9 ± 285.4	/	/
Fei Wang 2022 (P) [17]	48.10 ± 3.9	/	15.18 ± 2.8	/	5.3 ± 3.2	24.23 ± 2.7	18.93	262.0 ± 28.8	1103.2 ± 115.1	/	2 (5%) (2 infections)
Fei Wang 2022 (C) [17]	50.03 ± 4.9	/	20.33 ± 3.8	/	6.45 ± 2.9	19.93 ± 2.4	13.48	229.5 ± 26.8	979.8 ± 171.7	/	1 (2.5%) (1 case of abdominal pain treated with gastrointestinal decompression)
Harfouch 2022 (P) [16]	67.5 ± 19.5	/	20.4 ± 12.5	71.0 ± 10.9	29.0 ± 13.1	25.2 ± 6.0	−3.8 ± 11.6	/	/	/	5 (12.5%) (all IONM changes, 2 cases required two-stage surgery with temporary rod)
Harfouch 2022 (C) [16]	68.1 ± 14.9	/	25.0 ± 11.1	64.2 ± 11.5	36.2 ± 14.9	17.5 ± 9.4	−18.6 ± 10.1	/	/	/	0
Floccari 2021 (P) [18]	74.5 ± 15.2	39.6 ± 12.7	29.1 ± 8.6	66.6 ± 14.1	28.0 ± 16.0	22.6 ± 8.9	−5.5 ± 14.0	296.0 ± 64.0	825.0 ± 511.1	4.4 ± 1.0	11 (32.4%) (of whom 5 were IONM critical changes, 6 reoperations for mechanical failures or infections)
Floccari 2021 (C) [18]	70.8 ± 13.4	39.1 ± 10.6	21.3 ± 9.5	58.7 ± 10.3	27.6 ± 14.5	24.6 ± 9.7	−3.0 ± 12.1	286.3 ± 63.8	861.2 ± 583.4	4.6 ± 1.4	2 (5.9%) (1 reoperation for mechanical failure, 1 for infection)
Feng 2018 (P) [19]	57.6 ± 10.3	/	19.9 ± 1.6	63.9 ± 4.5	N (18): 21.3 ± 6.7 HyperK (3): 45.3 ± 5.5 HypoK (11): 7.3 ± 1.9	N (18): 28.4 ± 4.6 HyperK (3): 27.0 ± 2.0 HypoK (11): 18.4 ± 3.2	N (18): 7.1 ± 10.3 HyperK (3): −18.3 ± 2.5 HypoK (11): 11.1 ± 2.9	243.0 ± 12.0	952.0 ± 124.0	/	1 (3.1%) (hemopneumothorax)
Feng 2018 (C) [19]	56.1 ± 8.9	/	19.6 ± 2.9	65.2 ± 2.4	N (19): 23.7 ± 5.5 HyperK (4): 43.0 ± 1.4 HypoK (11): 8.0 ± 1.4	N (19): 24.4 ± 6.2 HyperK (4): 30.5 ± 1.3 HypoK (11): 11.5 ± 2.4	N (19): 0.7 ± 4.6 HyperK (4): −12.5 ± 1.3 HypoK (11): 3.5 ± 2.2	196.0 ± 10.0	772.0 ± 65.0	/	2 (6.1%) (surgical site infections)
Samdani,2015 (P) [20]	51.5 ± 8.6	47.3 ± 22.1	16.8 ± 6.3	67.1 ± 11.8	18.7 ± 13.0	21.8 ± 7.9	3.0 ± 11.6	277.4 ± 98.9	970.1 ± 566.5	5.3 ± 1.2	/
Samdani, 2015 (C) [20]	50.8 ± 8.1	54.5 ± 22.8	19.4 ± 7.0	61.8 ± 12.6	23.2 ± 12.3	22.8 ± 9.4	−0.4 ± 9.9	295.9 ± 136.4	778.9 ± 726.1	5.3 ± 1.2	/
Pizones 2015 (P) [23]	60.0 ± 9.9	/	17.4 ± 7.5	/	All cohort (43): 23.7 ± 13.7 N (27): 26 ± 6.1 HyperK (4): 52.2 ± 5.6 HypoK (12): 6.4 ± 2	All cohort: 22.0 ± 6.4 N (27): 25.9 ± 6 HyperK (4): 30.5 ± 8.5 HypoK (12): 21.3 ± 5.8	All cohort: −1.4 ± 12.5 N (27): 0.1 ± 6.9 HyperK (4): −21.7 ± 9.1 HypoK (12): 15.5 ± 7.5	258.0 ± 42.0	/	14	/
Pizones 2015 (C) [23]	60.4 ± 10.0	/	26.5 ± 8.0	/	All cohort (30): 23.5 ± 11.8 N (20): 24.2 ± 7.9 HyperK (3): 46 ± 3.4 HypoK (7): 9.8 ± 0.3	All cohort: 24.9 ± 9.9 N (20): 27.9 ± 7.5 HyperK (3): 38.5 ± 9.1 HypoK (7): 15 ± 7	All cohort: 1.0 ± 6.1 N (20): 3.4 ± 7.2 HyperK (3): −6.5 ± 5 HypoK (7): 5.6 ± 7.9	276.0 ± 54.0	/	6.7	/
Takahashi 2014 (P) [21]	52.5 ± 10.4	31.7 ± 13.2	18.4 ± 1.6	62.0 ± 2.5	11.3 ± 11.2	21.8 ± 1.7	10.5 ± 2.4	236.0 ± 13.0	1141.0 ± 150.0	/	/
Takahashi 2014 (C) [21]	51.5 ± 9.2	45.1 ± 12.3	17.8 ± 1.0	63.6 ± 2.5	13.0 ± 9.0	24.2 ± 1.9	11.2 ± 1.9	187.0 ± 9.0	745.0 ± 120.0	/	/
Halanski 2013 (P) [15]	59.0 ± 10.0	33.0 ± 19.0	9.0 ± 6.0	84.0 ± 9.0	20.0 ± 15.0	28.0 ± 8.0	8.0 ± 11.0	/	/	/	1 (5.9%) (pneumothorax)
Halanski 2013 (C) [15]	52.0 ± 8.0	41.0 ± 16.0	9.0 ± 4.0	83.0 ± 6.0	24.0 ± 12.0	25.0 ± 7.0	1.0 ± 10.0	/	/	/	1 (5.6%) (mispositioned screw that was removed)

**Table 3:** Reported complications stratified according to the modified Clavien–Dindo–Sink classification.

Author	Clavien–Dindo–Sink Grade I *n* (%) (Type)	Clavien–Dindo–Sink Grade II *n* (%) (Type)	Clavien–Dindo–Sink Grade III *n* (%) (Type)	Clavien–Dindo–Sink Grade I *n* (%) (Type)	Clavien–Dindo–Sink Grade IVb *n* (%) (Type)	Overall Complications *n* (%)
Fei Wang 2022 (P) [17]			2 (5%) (2 deep infections treated with surgical debridement and antibiotics)			2
Fei Wang 2022 (C) [17]			1 (2.5%) (1 case of abdominal pain treated with gastrointestinal decompression)			1 (2.5%)
Harfouch 2022 (P) [16]	3 (7.5%) (3 transient IONM changes that did not require staged surgery)		2 (5%) (2 IONM changes that required two-stage surgery with temporary rod)			5 (12.5%)
Harfouch 2022 (C) [16]						0
Floccari 2021 (P) [18]	4 (11.8%) (4 transient IONM changes that did not require staged surgery)		7 (20.6%) (1 IONM change that required two-stage surgery with temporary rod, 2 revisions for prominent implants, 1 revision for implant failure, 3 surgical debridements for surgical site infections)			11 (32.4%)
Floccari 2021 (C) [18]			2 (5.9) (1 reoperation for mechanical failure, 1 for infection)			2 (5.9%)
Feng 2018 (P) [19]	1 (3.1%) (1 hemopneumothorax treated with symptomatic and supportive treatments)					1 (3.1%)
Feng 2018 (C) [19]	2 (6.1%) (2 superficial surgical site infections treated with symptomatic and supportive treatments)					2 (6.1%)
Halanski 2013 (P) [15]	1 (5.9%) (pneumothorax)					1 (5.9%)
Halanski 2013 (C) [15]			1 (5.6%) (malpositioned screw that was removed)			1 (5.6%)

## Data Availability

The data presented in this study are available in article.

## References

[1] Guo X., Chau W.-W., Chan Y.-L., Cheng J.C.-Y. (2003). Relative anterior spinal overgrowth in adolescent idiopathic scoliosis. Results of disproportionate endochondral-membranous bone growth. J. Bone Jt. Surg. Br..

[2] Bodendorfer B.M., Shah S.A., Bastrom T.P., Lonner B.S., Yaszay B., Samdani A.F., Miyanji F., Cahill P.J., Sponseller P.D., Betz R.R. (2020). Restoration of Thoracic Kyphosis in Adolescent Idiopathic Scoliosis Over a Twenty-year Period: Are We Getting Better?. Spine.

[3] Watanabe K., Nakamura T., Iwanami A., Hosogane N., Tsuji T., Ishii K., Nakamura M., Toyama Y., Chiba K., Matsumoto M. (2012). Vertebral derotation in adolescent idiopathic scoliosis causes hypokyphosis of the thoracic spine. BMC Musculoskelet. Disord..

[4] Mladenov K.V., Vaeterlein C., Stuecker R. (2011). Selective posterior thoracic fusion by means of direct vertebral derotation in adolescent idiopathic scoliosis: Effects on the sagittal alignment. Eur. Spine J..

[5] Di Silvestre M., Lolli F., Bakaloudis G., Maredi E., Vommaro F., Pastorelli F. (2013). Apical vertebral derotation in the posterior treatment of adolescent idiopathic scoliosis: Myth or reality?. Eur. Spine J..

[6] Kim Y.J., Lenke L.G., Kim J., Bridwell K.H., Cho S.K., Cheh G., Sides B. (2006). Comparative analysis of pedicle screw versus hybrid instrumentation in posterior spinal fusion of adolescent idiopathic scoliosis. Spine.

[7] Lowenstein J.E., Matsumoto H., Vitale M.G., Weidenbaum M., Gomez J.A., Lee F.Y.-I., Hyman J.E., Roye D.P. (2007). Coronal and Sagittal Plane Correction in Adolescent Idiopathic Scoliosis: A comparison between all pedicle screw versus hybrid thoracic hook lumbar screw constructs. Spine.

[8] Bernstein P., Hentschel S., Platzek I., Hühne S., Ettrich U., Hartmann A., Seifert J. (2014). Thoracal flat back is a risk factor for lumbar disc degeneration after scoliosis surgery. Spine J..

[9] Hwang S.W., Samdani A.F., Tantorski M., Cahill P., Nydick J., Fine A., Betz R.R., Antonacci M.D. (2011). Cervical sagittal plane decompensation after surgery for adolescent idiopathic scoliosis: An effect imparted by postoperative thoracic hypokyphosis. J. Neurosurg. Spine.

[10] Demura S., Yaszay B., Carreau J.H., Upasani V.V., Bastrom T.P., Bartley C.E., Newton P.O. (2013). Maintenance of Thoracic Kyphosis in the 3D Correction of Thoracic Adolescent Idiopathic Scoliosis Using Direct Vertebral Derotation. Spine Deform..

[11] Faldini C., Viroli G., Barile F., Manzetti M., Ialuna M., Traversari M., Vita F., Ruffilli A. (2023). One stage correction via the Hi-PoAD technique for the management of severe, stiff, adolescent idiopathic scoliosis curves > 90°. Spine Deform..

[12] Ponte A., Orlando G., Siccardi G.L. (2018). The True Ponte Osteotomy: By the One Who Developed It. Spine Deform..

[13] Moher D., Liberati M., Tetzlaff J., Altman D.G., PRISMA Group (2009). Preferred reporting items for systematic reviews and meta-analyses: The PRISMA statement. PLoS Med..

[14] Sterne J.A.C., Hernán M.A., Reeves B.C., Savović J., Berkman N.D., Viswanathan M., Henry D., Altman D.G., Ansari M.T., Boutron I. (2016). ROBINS-I: A tool for assessing risk of bias in non-randomised studies of interventions. BMJ.

[15] Halanski M.A., Cassidy J.A. (2013). Do multilevel Ponte osteotomies in thoracic idiopathic scoliosis surgery improve curve correction and restore thoracic kyphosis?. J. Spinal Disord. Tech..

[16] Harfouch E.B., Bunyan R.F., Al Faraidy M., Alnemari H.H., Bashir S. (2022). Ponte osteotomies increase risk of intraoperative neuromonitoring alerts in adolescent idiopathic scoliosis surgery. Surg. Neurol. Int..

[17] Wang F., Chen K., Ji T., Ma Y., Huang H., Zhou P., Wei X., Chen Z., Bai Y. (2022). Do hypokyphotic adolescent idiopathic scoliosis patients treated with Ponte osteotomy obtain a better clinical efficacy? A preliminary retrospective study. J. Orthop. Surg. Res..

[18] Floccari L.V., Poppino K., Greenhill D.A., Sucato D.J. (2021). Ponte osteotomies in a matched series of large AIS curves increase surgical risk without improving outcomes. Spine Deform..

[19] Feng J., Zhou J., Huang M., Xia P., Liu W. (2018). Clinical and radiological outcomes of the multilevel Ponte osteotomy with posterior selective segmental pedicle screw constructs to treat adolescent thoracic idiopathic scoliosis. J. Orthop. Surg. Res..

[20] Samdani A.F., Bennett J.T., Singla A.R., Marks M.C., Pahys J.M., Lonner B.S., Miyanji F., Shah S.A., Shufflebarger H.L., Newton P.O. (2015). Do Ponte Osteotomies Enhance Correction in Adolescent Idiopathic Scoliosis? An Analysis of 191 Lenke 1A and 1B Curves. Spine Deform..

[21] Takahashi J., Ikegami S., Kuraishi S., Shimizu M., Futatsugi T., Kato H. (2014). Skip pedicle screw fixation combined with Ponte osteotomy for adolescent idiopathic scoliosis. Eur. Spine J..

[22] Tanida S., Masamoto K., Tsukanaka M., Futami T. (2023). No short-term clinical improvement and mean 6° of thoracic kyphosis correction using limited-level Ponte osteotomy near T7 for Lenke type 1 and 2 adolescent idiopathic scoliosis: A preliminary study. J. Pediatr. Orthop. B.

[23] Pizones J., Sánchez-Mariscal F., Zúñiga L., Izquierdo E. (2015). Ponte osteotomies to treat major thoracic adolescent idiopathic scoliosis curves allow more effective corrective maneuvers. Eur. Spine J..

[24] Dodwell E.R., Pathy R., Widmann R.F., Green D.W., Scher D.M., Blanco J.S., Doyle S.M., Daluiski A., Sink E.L. (2018). Reliability of the Modified Clavien-Dindo-Sink Complication Classification System in Pediatric Orthopaedic Surgery. JBJS Open Access.

[25] Kuklo T.R., Potter B.K., Schroeder T.M., O’brien M.F. (2006). Comparison of manual and digital measurements in adolescent idiopathic scoliosis. Spine.

[26] Mirzashahi B., Moosavi M., Rostami M. (2020). Outcome of Posterior-Only Approach for Severe Rigid Scoliosis: A Retrospective Report. Int. J. Spine Surg..

[27] Koerner J.D., Patel A., Zhao C., Schoenberg C.B., Mishra A., Vives M.J., Sabharwal S. (2014). Blood loss during posterior spinal fusion for adolescent idiopathic scoliosis. Spine.

[28] Dong Y., Tang N., Wang S., Zhang J., Zhao H. (2021). Risk factors for blood transfusion in adolescent patients with scoliosis undergoing scoliosis surgery: A study of 722 cases in a single center. BMC Musculoskelet. Disord..

[29] Yoo J.S., Ahn J., Karmarkar S.S., Lamoutte E.H., Singh K. (2019). The use of tranexamic acid in spine surgery. Ann. Transl. Med..

[30] Iorio J., Bennett J.T., Orlando G., Singla A., Dakwar E., Bonet H., Samdani A.F. (2013). Does Amicar affect blood loss in patients with adolescent idiopathic scoliosis treated with pedicle screws and Ponte osteotomies?. Surg. Technol. Int..

[31] Baird E.O., McAnany S.J., Lu Y., Overley S.C., Qureshi S.A. (2015). Hemostatic Agents in Spine Surgery: A Critical Analysis Review. JBJS Rev..

[32] Lee C.S., Hwang C.-J., Lee D.-H., Cho J.H., Park S. (2023). Risk Factors and Exit Strategy of Intraoperative Neurophysiological Monitoring Alert During Deformity Correction for Adolescent Idiopathic Scoliosis. Glob. Spine J..

[33] Buckland A.J., Moon J.Y., Betz R.R., Lonner B.S., Newton P.O., Shufflebarger H.L., Errico T.J., Harms Study Group (2019). Ponte Osteotomies Increase the Risk of Neuromonitoring Alerts in Adolescent Idiopathic Scoliosis Correction Surgery. Spine.

[34] Feng B., Qiu G., Shen J., Zhang J., Tian Y., Li S., Zhao H., Zhao Y. (2012). Impact of multimodal intraoperative monitoring during surgery for spine deformity and potential risk factors for neurological monitoring changes. J. Spinal Disord. Tech..

[35] Perdriolle R., Le Borgne P., Dansereau J., de Guise J., Labelle H. (2001). Idiopathic scoliosis in three dimensions: A succession of two-dimensional deformities?. Spine.

[36] Parvaresh K.C., Osborn E.J., Reighard F.G., Doan J., Bastrom T.P., Newton P.O. (2017). Predicting 3D Thoracic Kyphosis Using Traditional 2D Radiographic Measurements in Adolescent Idiopathic Scoliosis. Spine Deform..

[37] Monazzam S., Newton P.O., Bastrom T.P., Yaszay B., Harms Study Group (2013). Multicenter Comparison of the Factors Important in Restoring Thoracic Kyphosis During Posterior Instrumentation for Adolescent Idiopathic Scoliosis. Spine Deform..

